# *Leptospira* seroprevalence and associated risk factors among cattle in Bor County, South Sudan

**DOI:** 10.1371/journal.pone.0325492

**Published:** 2025-06-06

**Authors:** Kitale Estella John Kasiano, Lordrick Alinaitwe, Walter Okello, Tubihemukama Methodius, Christopher Joshua Aturinda, Ashiraf Lubega, Esther Sabbath Frazer Togo, Peter Micheal Marin, David Kal Onafruo, Ambrose Samuel Jubara, Savino Biryomumaisho, Clovice Kankya

**Affiliations:** 1 College of Veterinary Medicine, Animal Resources and Biosecurity, Makerere University, Kampala, Uganda; 2 College of Veterinary Science, University of Bahr El Ghazal, Wau, South Sudan; 3 International Institute of Tropical Agriculture, Kampala, Uganda; Universita degli Studi di Parma, ITALY

## Abstract

Leptospirosis is a bacterial zoonotic disease that is distributed globally. In livestock, leptospirosis often presents as a subclinical disease that results in significant reproductive and production losses, which could in turn have detrimental economic consequences, particularly in countries like South Sudan that rely on livestock farming for livelihood. Leptospirosis often presents as a subclinical disease in which case the animal may be a maintenance host for a specific serovar. Recent increases in unexplained abortions have prompted us to investigate *Leptospira* exposure and associated risk factors among cattle in Bor County South Sudan. A cross-sectional study was conducted between 22^nd^ January to 15^th^ February 2023. Blood samples were collected from 357 cattle in four of the six cattle camps in the County at that time. Seropositivity was determined by detecting anti-*Leptospira* antibodies in the serum samples by microscopic agglutination test (MAT) based on a panel of 12 serovars representing 12 serogroups. Data on risk factors were obtained using pre-tested questionnaires administered to the owner or herdsman of each sampled herd. Of the 357 cattle sampled, 66.95% (95% CI = 61.91–71.62) were seropositive (cut-off titer ≥100). Seventy-six of the seropositive cattle (21.65%) had MAT titer ≥800, indicating a probable recent infection at the time of sampling. The most prevalent serogroups were *L. borgpetersenii* Tarassovi (59.83%) and *L*. *borgpetersenii* Ballum (17.38%). In the robust Poisson regression model, only the age of cattle was a significant risk factor to *Leptospira* seroprevalence. The prevalence in adult cattle was 1.43 times higher than in young ones (95% CI 1.09–1.92; P-value = 0.012). The extremely high seroprevalence indicates that leptospirosis may be endemic in cattle in South Sudan, and potentially one of the etiologies for the recently increasing abortion reports. This may require confirmation of the infection status among the aborting cattle.

## Introduction

*Leptospira* refers to a genus of spirochete bacteria that cause leptospirosis, a globally distributed zoonotic disease [1]. To date, over 69, *Leptospira* species and over 300 serovars have been documented [2], with several of these serovars reported as regionally endemic or adapted to certain animal reservoirs [3]. Rodents, and various wild, and domestic animals have also been shown to maintain several *Leptospira* serovars. For example, Bratislava in pigs, Hardjo in cattle and Icterohaemorrhagiae in the brown Norwegian rat (*Rattus norvegicus)* [4]. Infection in humans and other susceptive animal hosts results from contact with infected urine or contaminated water and soil [5,6].

The annual global incidence of leptospirosis was estimated at 1.03 million human cases with 58,900 deaths in 2015 [1]. Furthermore, the Pan American Health Organization/World Health Organization in 2016 estimated about 750,000 leptospirosis cases in Africa per year, and that the disease burden is higher in limited-resource settings of tropical and subtropical regions where the disease is regarded as endemic [7]. In livestock, *Leptospira* infection is usually associated with reproductive and production losses resulting from abortions, treatment costs, stunting in young animals, reduced milk yield, and even death [8]. This may mean detrimental economic outcomes, especially in populations that depend on livestock for livelihood, notably in low and middle-income countries [9].

In South Sudan, seroprevalence of 63.5% was reported in 1989 in domesticated animals, including cattle, with serovars Tarassovi, Hebdomadis, Bataviae, Pomona, Icterohaemorrhagiae, Grippotyphosa and Autumnalis [10]. Although no cases of leptospirosis have been reported in cattle in South Sudan since then, there is growing evidence of increased livestock production and reproductive issues, notably abortions [11]. Only 10% of these abortions could be attributed to brucellosis, leaving a great proportion unexplained. Recent climatic changes in South Sudan have resulted in intensified rainfall and flooding. These factors, coupled with the nomadic way of life in most South Sudan communities, communal grazing, and sharing of water sources such as rivers, and streams between livestock and humans present an ideal epidemiological setting for the maintenance and spread of *Leptospira* infections in both livestock and humans. In the current study, we investigated *Leptospira* seroprevalence and associated risk factors among cattle from pastoral communities of Bor County.

## Materials and methods

### Study area

Bor is part of Jonglei state in the central region of South Sudan on the Eastern bank of the White Nile with a human population of about 372,043 according to Sudan’s fifth population census [12]. The County is mostly settled by agropastoral nomadic communities who keep cattle by freely ranging on communal land called camps. It is among the major cattle-keeping states of South Sudan that experiences extremes of dry and wet climates with the highest rainfall received between May and August and precipitation of 99 mm to 120 mm [13].

### Study design and sampling strategy

A cross-sectional study was conducted among the pastoral communities of Bor County, Jonglei State from 22^nd^ January to 15^th^ February 2023.

Cattle held in four of the six camps present in Bor County at the time were sampled. The sampled camps were Malualchat, Kuada, Thoony and Pabial. Access to other camps was not possible due to insecurity caused by cattle raids at the time. All cattle younger than six months, and those that had come into Bor County from other locations within the last year, were excluded since they could have been exposed outside of Bor. About 30% of the cattle in each of the small herds (20–30 cattle) and 40% of the large herds (31–67 cattle) were randomly sampled. While we sampled all the consenting herd-owners, individual cattle from each herd were selected randomly.

### Sample size determination

Sample size of 355 was estimated in Epitools Epidemiological Calculators [14], based on 63.5% seroprevalence from an earlier report in Upper Nile state [10], confidence level of 0.95, and an error margin of 5%. Since the cattle camps had uniform geographical and demographic characteristics, they were all considered a homogeneous population. The number of cattle sampled in each of the four camps was determined as a product of the sample size estimate and the proportion of cattle in the camp to the total estimated population of 1050 cattle in the four camps at the time, with the following distribution: 150 in Kuada, 300 in Malualchat, 250 in Pabial and 350 in Thoony respectively. The number of respondents to the questionnaire corresponded with the number of herd-owners consenting to participate in the study.

### Sample collection

About 5–8 mL of blood was collected from the jugular vein, into clot-activated serum separator tubes (BD Vacutainer Ref:367958). Samples were kept on ice until delivered to a laboratory in Bor State Hospital. At the laboratory, sera were extracted into 1.5mL cryogenic tubes following centrifugation at a speed of 3000*g* for 3 minutes. The extracted sera were frozen at −20°C and, transported by air under ice to the College of Veterinary Medicine Animal Resources and Biosecurity (CoVAB), Makerere University, Kampala, Uganda, and immediately frozen at −20°C on arrival.

For each sampled animal, the age was estimated empirically and or by dentition [15], with cattle approximately less than two years scored young. The body condition was scored as poor, good or very good based on examination of fat cover over the cattle’s body [16].

### Collection of information on risk factors

A pre-tested questionnaire (S1 Text) was used to collect herd demographic data, farm health history, environmental data such as the presence of wildlife reservoirs, and climatic factors. The questionnaire was digitized in Open Data Kit (ODK version v2021.2.3) and administered to herdsmen and/or owners of the herds by the researcher following verbal consent, which was captured in the questionnaire as well. Interviews were administered in English and Arabic. In a few instances, a native pre-trained research assistant translated directly into the local Dinka language.

### Laboratory analysis

Microscopic agglutination test (MAT) was used to determine the anti-*Leptospira* antibodies according to OIE standards [17]. The MAT panel comprised 12 serovars representing 12 serogroups sourced from the World Organization of Animal Health (WOAH) reference laboratory for leptospirosis, Netherlands (Table 1). The panel was selected to include serovars which were described as prevalent in the previous study in the Upper Nile province of South Sudan [10] and neighboring East African countries [18]. Briefly, seven-day-old live *Leptospira* cultures were used to screen the serum samples at an initial dilution of 1:50. Those with a positive reaction were then titrated in a two-fold serial dilution to find the end titer. Sera with a titer ≥1:100 were considered positive [17].

**Table 1:** Strains of *Leptospira* species used as live antigens in the MAT.

Genomospecies	Serogroup	Serovar	Strain
*L. interrogans*	Icterohaemorrhagiae	Icterohaemorrhagiae	RGA
	Australis	Australis	Ballico
	Pomona	Pomona	Pomona
	Canicola	Canicola	Strain Hond Utrecht IV
	Hebdomadis	Hebdomadis	Hebdomadis
*L. kirschneri*	Autumnalis	Butembo	Butembo
	Grippotyphosa	Grippotyphosa	Duyster
*L. borgpetersenii*	Pyrogenes	Nigeria	Vom
	Tarassovi	Tarassovi	Perepelitsin
	Sejroe	Sejroe	M84
	Ballum	Kenya	Njenga
*L.weilli*	Celledoni	Celledoni	Celledoni

### Data management and analysis

The data were retrieved from the ODK and entered in Microsoft Excel, cleaned, and checked for completeness and consistency. R statistical package version 4.3.0 was used for analysis. *Leptospira* seroprevalence was calculated using “EpiR” using the “epi.prev” function. Differences in *Leptospira* seroprevalence by cattle breed, sex, body condition score, grazing environment, and herd health history were described and analyzed by Chi‐square test (univariable analysis). Exposure variables were entered in robust Poisson regression model if the univariable p‐value was ≤ 0.2 and kept in the model if the Likelihood ratio test was statistically significant (p ≤ 0.05). A robust Poisson regression with “log” as the link function was considered given the high prevalence of the outcome (*Leptospira* seroprevalence). There was no variation in the outcome between the camps, and clustering by herd was reasoned out by the fact that all cattle within a camp were grazed together in communal fields. For the final model, the backward selection was implemented using the function “step” in the package “MASS”, and the outcome of interest was *Leptospira* seroprevalence in cattle. The following interaction terms were explored; Sex:Age, Age:Breed. Collinearity in the model was ruled out by measuring the variance inflation factor using the function “vif” from the package “Car”, and model fitness was assessed by measuring the dispersion, residual degrees of freedom and inspecting residue plots.

### Ethical considerations

Written approval to collect cattle samples was granted by the Director General, Ministry of Livestock and Fisheries, Juba, South Sudan (RSS/MLF/DVS/39/023) after a review of the research activity, inclusive of animal welfare, by their committee board. Additionally, verbal consent for taking samples from the cattle and for responding to the questionnaires was obtained, witnessed by cattle camp leader and recorded digitally in the questionnaire (S1 Text). This was deemed sufficient as the information gathered by the questionnaire was perceived by the camps’ leaders and the researchers as neither presenting foreseeable breaches of respondents’ privacy and rights nor posing any direct health risks. Permission to import serum samples into Uganda was obtained from the Ministry of Agriculture Animal Industry and Fisheries (MAAIF) Uganda (DAH2023010300014).

## Results

### Demographics of the sampled cattle and respondent attributes

The number of cattle sampled per camp were 55 (15.41%), 99 (27.43%), 83 (23.25%), and 120 (33.61%) from Kuada, Malualchat, Pabial and Thoony respectively. Most of the cattle sampled were female (70.87%), adult (64.15%), and in good body condition (66.11%). All the cattle except the Lugbara were breeds local to South Sudan (Table 3). The respondents to the questionnaire were mainly male (92.44%) and either owners, relatives, or herdsmen who had stayed with the herd for at least one year.

**Table 2:** The proportion of reactions and titer levels of Serogroup-specific anti-*Leptospira* antibodies measured by microscopic agglutination test among cattle in Bor County, South Sudan.

MAT titre									
Serovar	100	200	400	800	1600	3200	6400	12800	Npos (%)
Tarassovi	37	72	46	41	8	3	1	2	210 (59.82)
Kenya	20	25	8	5	3	0	0	0	61 (17.38)
Australis	4	7	6	1	2	3	0	1	24 (6.84)
Butembo	17	1	0	0	0	0	0	0	18 (5.13)
Sejroe	8	3	0	1	0	0	0	0	12 (3.42)
Grippotyphosa	7	1	1	2	0	0	0	0	11 (3.13)
Hebdomadis	5	1	2	1	0	1	0	0	10 (2.85)
Pomona	0	1	2	0	1	0	0	0	4 (1.14)
Nigeria	1	0	0	0	0	0	0	0	1 (0.28)
Icterohaemorrhagiae	0	0	0	0	0	0	0	0	0 (0.00)
Celledoni	0	0	0	0	0	0	0	0	0 (0.00)
Canicola	0	0	0	0	0	0	0	0	0 (0.00)
Total	99	111	65	51	14	7	1	3	351

Npos; number of seropositive reactions (including multiple reactions)

**Table 3:** Demography and factors associated with *Leptospira* seroprevalence among cattle in Bor County, South Sudan (*N* = 357).

Variable	Level	Number	%Frequency	%Pos	Prevalence ratio (95% CI)	P-value
Herd size	Small	75	21.01	61.33	0.9 (0.64-1.22)	0.504
	Large	282	78.99	68.44	Ref	
Sex	Male	104	29.13	55.77	0.78 (0.58-1.04)	0.098
	Female	253	70.87	71.54	Ref	
Age	Young	128	35.85	52.34	0.7 (0.52-0.91)	0.012
	Adult	229	64.15	75.11	Ref	
Breed	Lugbara	36	10.08	86.11	1.36 (0.91-1.98)	0.118
	Murle	5	1.4	0	NA	
	Nuer	85	23.81	67.06	1.06 (0.78-1.43)	0.705
	Dinka	231	64.71	63.2	Ref	
Body condition	Poor	92	25.77	63.04	0.93 (0.68-1.25)	0.635
	Good	236	66.11	67.8	Ref	
	Very good	29	8.12	72.41	1.07 (0.65-1.64)	0.77
Mixed grazing	Goats and sheep	174	48.74	58.62	0.72 (0.45-1.25)	0.732
	None	162	45.38	74.07	0.92(0.57-1.58)	0.218
	Dogs	21	5.88	80.95	Ref	
Rodents in the grazing field	Infrequent	66	18.49	75.76	1.17 (0.84-1.58)	0.333
	Frequent	291	81.51	64.95	Ref	
Wildlife in the grazing field	Infrequent	244	68.35	66.8	0.99 (0.76-1.31)	0.96
	Frequent	113	31.65	67.26	Ref	
Grazing field	Swamp	304	85.15	66.12	0.79 (0.55-1.19 )	0.237
	Open grassland	17	4.76	47.06	0.56 (0.24-1.17)	0.151
	Forest	36	10.08	83.33	Ref	
Drinking water	Stagnant source	328	91.88	65.55	0.79 (0.53-1.23)	0.279
	River	29	8.12	82.76	Ref	
Recent floods	Yes	328	91.88	67.99	0.81 (0.47-1.30)	0.42
	No	29	8.12	55.17	Ref	
Recent abortion	Yes	314	87.96	66.88	0.99 (0.68-1.49)	0.966
	No	43	12.04	67.44	Ref	
Cause of recent deaths	Non-infectious	118	33.05	68.64	1.04 (0.79-1.35)	0.783
	Infectious	239	66.95	66.11	Ref	

*NA- the five cattle of the Murle breed were left out of the risk factor analysis. Ref - the reference category within the variable which the others have been compared.*

### Prevalence of anti-*Leptospira* antibodies

Of the 357 cattle tested, 239 (66.95%, 95% CI = 61.91–71.62) were seropositive (cut-off titer ≥100). Most seropositive cattle reacted to serogroups Tarassovi (59.83%), and Ballum Serovar Kenya (17.38%) (Table 2). Positivity to multiple *Leptospira* serovars was detected in 31.92%, (n = 112, 95% CI = 27.25–36.96) of the cattle. Seventy-six of the seropositive cattle (21.65%) had MAT titer ≥800, indicating a probable recent infection at the time of sampling.

### Risk factors

In the univariable analysis, age, breed, sex, grazing with small ruminants, and grazing in swampy fields, had a p‐value of ≤0.2 and thus were considered in the robust Poisson regression model (Table 3). In the final model, only the age of cattle was a significant risk factor. The prevalence in adult cattle was 1.43 times higher than in young ones (95% CI 1.09–1.92; P-value = 0.012). The model had a dispersion of 0.33, and the residual plots did not show any pattern of misfit.

## Discussion

A high *Leptospira* seroprevalence was detected among cattle in Bor County, South Sudan, with serovars Tarassovi (serogroup Tarassovi) and Kenya (Ballum) being highly prevalent. *Leptospira* seroprevalence was significantly associated with age of the cattle. Although globally cattle are known to be host for *L. borgpetersenii* serovar Hardjo (Sejroe serogroup), in this study low seroprevalence of the serovar Sejroe serogroup was noted. Comparably high *Leptospira* seroprevalence and similar serogroups were reported earlier in the Upper Nile region in 1989 [10], and recently in Western Bahr El Ghazal region [19]. This may imply that leptospirosis is endemic in South Sudan and that cattle may be a historical *Leptospira* carrier. The high seropositivity among the cattle in this study could be attributed to various factors like difference in climate, rainfall patterns, geographical location, husbandry and the nomadic way of life of the cattle keepers in Bor County. This involves movement of large herds of cattle between places while sharing the same water points and grazing land which increases the risk of exposure to *Leptospira* [20]. Moreover, the months of January and February during which the study was conducted represent the middle of a dry spell in South Sudan that is characterized by water scarcity, further intensifying the above practices. The high seroprevalence obtained in the current study also corroborates with the findings of other studies conducted elsewhere in East Africa. For example, a seroprevalence ranging between 19.27% and 27.8% has been found among cattle in Uganda [21,22]. A study conducted in Nyandarua and Turkana, Kenya reported a prevalence of 49% and 44% in cattle respectively [23]. South Sudan neighbors Uganda in the South and Kenya in the Southeast, in areas of these countries that are occupied by pastoralists. It is thus possible that the movement of animals across these borders during trade, and refugee settlement contributes to the spread of the disease in either country. Moreover, the same *Leptospira* serogroups, including Tarassovi that were found highly prevalent in the current study have been reported among domestic animals in the rest of East Africa [21,24–26]. No cattle reacted to serovars Icterohaemorrhagiae, Canicola, and Celledoni. Serovars Canicola, Icterohaemorrhagiae, and Celledoni are associated with cases of leptospirosis in dogs [27], yet a low number of respondents reported keeping dogs, possibly reducing exposure to these serovars.

Finding high antibody titers (≥800) indicates that these cattle were probably infected at the time of sampling. Leptospires have been shown to persist in kidneys of infected animals for weeks to years, with the possibility of shedding the bacteria in urine. Individuals who are in close contact with cattle including farmers, veterinarians, herdsmen and slaughterhouse workers may be at particularly high risk of acquiring *Leptospira* infection. It is crucial that renal *Leptospira* carriage or urinary shedding is confirmed among livestock in South Sudan, and exposure in human populations be determined.

The association of adult cattle with *Leptospira* seropositivity as observed in the current study has been consistently reported elsewhere in the world [21,28] and has been attributed to the prolonged exposure to contaminated environmental sources among adult animals, having lived there longer than their young counterpart [21]. The lack of association between *Leptospira* seropositivity and mixed grazing may imply that the common *Leptospira* serovars in cattle are less adapted to infect small ruminants, such that the latter have a limited role in maintenance and shedding of *Leptospira*. Additionally, grazing cattle together with goats and sheep can reduce the overall density of cattle in the grazing area, consequently decreasing the probability of direct contact among cattle or with contaminated environments.

## Conclusion

In conclusion, cattle in Bor are highly exposed to pathogenic *Leptospira* species, especially of the Tarassovi and Ballum serogroups, indicating that leptospirosis may be endemic in cattle in South Sudan, and potentially one of the etiologies for the recently increasing abortion reports. This contribution of leptospirosis to cases of abortion should be confirmed by such tests as PCR and culturing. Potential *Leptospira* carriers including livestock and small mammals and pathways for spread of infection need to be investigated to inform local prevention and control strategies.

## Supporting information

S1 Text The questionnaire that was developed to collect data on risk factors. (DOCX)

S2 Text The raw csv file of the dataset used for analysis in this study. (XLSX)
