# Supplementary material for: Leptospira seroprevalence and associated risk factors among cattle in Bor County, South Sudan
Source: PLoS One. 2025 Jun 6;20(6):e0325492. doi: 10.1371/journal.pone.0325492 (PMC12143547; doi:10.1371/journal.pone.0325492)
Supplement: S1 Text — (DOCX) [file pone.0325492.s001.docx]

**Annex I Questionnaire and Consent form for investigation of *Leptospira* exposure in Cattle**

**
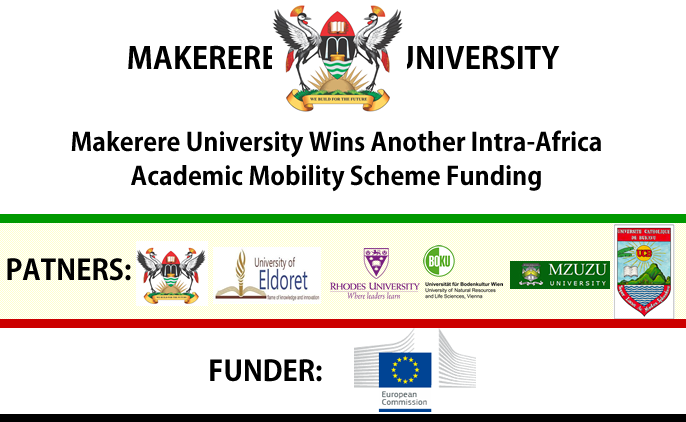
**

**MAKERERE UNIVERSITY**

**College of Veterinary Medicine, Animal Resources and Biosecurity (COVAB).**

**TITLE: *LEPTOSPIRA* SEROPREVALENCE AND ASSOCIATED RISK FACTORS AMONG CATTLE IN JONGLEI STATE, SOUTH SUDAN**

**Data form to be filled during sample collection.**

Date of visit………………………………………………………………………

Name of interviewer……………………………………………………………….

Name of cattle camp visited………………………………………………………….

**INTRODUCTION**

**Dear Respondent,**

I am Kitale Estella John, an MSc student from Makerere University, conducting this research and my colleague ……………………………………………………. We are visiting your cattle camp today as part of effort to improve understanding of leptospirosis- as a zoonotic disease of public health importance and also that causes reproductive and productive failures in Jonglei State South Sudan.

You have been selected to participate in this study. The findings of this study will be used

strictly for academic purposes and will be treated with great confidentiality. Your active participation and accurate information will help in the disease control in South Sudan.

Thank you for choosing to participate in this study.

**Participant`s Consent**

I……………………………………… voluntarily agree to participate in this research study, I understand that the information I give here will be treated with confidentiality. I have been explained to the purpose, and had chance to ask questions about the study. I also understand that in any report on the results of this research, my identity will remain anonymous.

Respondent signature…………………………… Date ………………………………

Interviewer’s signature……………………………Date……………………………………

**SECTION1: Demographic characteristics of the herd and herder.**

| 1.1 Sex of respondent | Male  Female  |
| --- | --- |
| 1.2 Age of respondent | **…………………. years** |
| 1.3 Which level of education did you stop at? | No formal education 🞏 Primary level 🞏 Secondary level 🞏 Tertiary level  |
| 1.4 Breed of the cattle | Crossbreed  Dinka  Nuer  others (Specify) |
| 1.5 Production system used | Semi intensive  Free range  Nomadic pastoralism  |
| 1.6 How do you relate to these animals? | Owner  herdsmen  Relative  |
| 1.7 How long have you been with this herd? | Less than 1 month  2-6 months  7-12 months  Over a year |
| 1.8 How many cattle do you have in this herd we have just picked blood from? | Less than 50  50 -100  more than 100  |
| 1.9 Aside cattle, which other type of animals do you keep? | None  Goats  Sheep  Pigs  Dogs  Cats Donkeys/Horses Oxen  Others (Specify)………………… |
| 1.10 How often do the other animals you keep graze with your cattle? | Daily  A few times a week  May not in a week but not for a month  A few times a times a year  Not in the past one year  |

**SECTION 2: PRESENCE OF THE RESERVOIR**

| 2.1 How often do you see rats or mice near your herd/camp? | Daily 🞏 At least once a week 🞏 May not in a week but not for a month   Can`t take a year without 🞏 Never see  |
| --- | --- |
| 2.2 How often do you see signs of rodent dropping or feeding traces in the vicinity of the camp / grazing area? | Daily 🞏 At least once a week 🞏 May not in a week but not for a month   Can`t take a year without 🞏 Never see  |
| 2.3 How often do you see wild animals in the place you graze your animals? | Daily  At least once a week  May not in a week but not for a month   Can`t take a year without  Never see  |

**SECTION 3: ENVIRONMENTAL FACTORS**

| 3.1 Description of the current grazing land | Paddock Open grassland  Swamp  Forest   Wildlife Park  |
| --- | --- |
| 3.2 Other economic activities done in the vicinity | Food Crop gardens  Fishing  Sand mining  wood cutting  Others (specify)……………. |
| 3.3 How often do you take your animals to graze outside this Payam? | Daily  At least once a week  May not in a week but not for a month   Can`t take a year without  Not in the last one year  Never  |
| 3.4 How do the other places you take your animals to graze look like? | Paddock  Open grassland  Swamp  Forest   Wildlife Park  (Tick many as may apply) |
| 3.5 Do other farmers also graze in the same places as you graze your animals? | Yes  No  |
| 3.6 What kind of water source do your animals commonly drink form? | Open well  River/stream  Pond  Swamp  Lake Puddles/ Stagnant water  Communal Reservoir  Others (specify)……………………... |
| 3.7 Do other farmers also bring their animals to drink from the same sources as you usually take your animals? | Yes  No  |

**SECTION 4: Climatic factors**

| 4.1 When was the last time you experienced rain on three consecutive days in your grazing areas? | Less than a week ago  a month ago  2-6 months ago  Almost a year ago  Over a year ago  Never  |
| --- | --- |
| 4.2 When was the last time you experienced flooding in your grazing areas? | Less than a week ago  a month ago  2-6 months ago  Almost a year ago  Over a year ago  Never  |

**SECTION 5: Reproduction and Production losses factors**

| 5.1 When did you last experience an abortion in cattle on your herd? | Less than a week ago  a month ago  2-6 months ago  Almost a year ago  Over a year ago  Never  |
| --- | --- |
| 5.2 About how old was the fetus at the time it aborted? | Less than 3 months  3-6 months  Over 6 months  |
| 5.3When did you last hear another farmer in your area/ payam/camp complain of an abortion in their herd? | Less than a week ago  a month ago  2-6 months ago  Almost a year ago  Over a year ago  Never  |
| 5.4 How satisfied with the amount of milk that you currently get from all your milking cows? | Very satisfied  Fairly satisfied  Not sure  unsatisfied  |
| 5.5 When did you last have a dead animal on your farm? | Less than a week ago  a month ago  2-6 months ago  Almost a year ago  Over a year ago  Never  |
| 5.6 How old was the animal? | Less than 6 months old  7-12 months old  1-3 years older  Over 3 years old  |
| 5.7 What do you think was the cause of its death? | Disease  Accident  Poisoning  Bewitched  None of these  |
| 5.8 Did the last dead animal present with any of the following symptoms before death? | Yellowing of eyes  Red urine  High temperature Excessive thirst  |

**Annex II Animal checklist**

**ANIMAL DATA FORM**

**Date………………… Observer/sample collector……………………………………………**

**Herd No……………... Herd size …………………………. Contact ……………...**

**Demographics of sampled cattle**

| Animal ID | Sex | Age | Breed | Location | Body score | | |
| --- | --- | --- | --- | --- | --- | --- | --- |
|  |  |  |  |  | P | G | VG |
| 1 |  |  |  |  |  |  |  |
| 2 |  |  |  |  |  |  |  |
| 3 |  |  |  |  |  |  |  |
| 4 |  |  |  |  |  |  |  |
| 5 |  |  |  |  |  |  |  |
| 6 |  |  |  |  |  |  |  |
| 7 |  |  |  |  |  |  |  |
| 8 |  |  |  |  |  |  |  |
| 9 |  |  |  |  |  |  |  |
| 10 |  |  |  |  |  |  |  |

**Note:** Breed can be Dinka (D), Nuer (N), Others (Specify). Age will be either young or Adult.
